# Endoscopic ultrasound-guided radiofrequency ablation of pancreatic insulinoma

**DOI:** 10.1590/0102-67202025000052e1921

**Published:** 2026-07-06

**Authors:** Silvia Johanna LOZADA-CALLE, Mateus Pereira FUNARI, Matheus de Oliveira VERAS, Rayssa Kethlyn Alves de CAMPOS, Jurandir Batista da CRUZ, Wanderley BERNARDO, Eduardo Guimarães Hourneaux de MOURA

**Affiliations:** 1 Universidade de São Paulo, Faculty of Medicine, Hospital das Clínicas, Endoscopy Unit – São Paulo (SP), Brazil.; 2 Universidade de São Paulo, Faculty of Medicine – São Paulo (SP), Brazil.; 3 Brazilian Medical Association, Medical Guidelines Program – São Paulo (SP), Brazil.; 4 Universidade de São Paulo, Faculty of Medicine, Graduate Program in Gastroenterology – São Paulo (SP), Brazil.

**Keywords:** Insulinoma, Pancreatic Neoplasms, Radiofrequency Ablation, Insulinoma, Neoplasias Pancreáticas, Ablação por Radiofrequência

## Abstract

**Background:**

Insulinoma is a rare pancreatic neuroendocrine tumor (pNET) arising from beta cells, leading to excessive insulin secretion and life-threatening hypoglycemia. While surgical resection remains the gold standard, endoscopic ultrasound-guided radiofrequency ablation (EUS-RFA) has emerged as a minimally invasive alternative, particularly for patients unfit for surgery.

**Aims:**

A meta-analysis was performed according to PRISMA (Preferred Reporting Items for Systematic Reviews and Meta-Analysis) guidelines to compare the efficacy and safety of EUS-RFA and surgery for pancreatic insulinomas.

**Methods:**

Systematic searches were conducted in PubMed, Cochrane Library, Embase, and Scopus, using MeSH terms related to insulinoma, RFA, and surgery. Eligible studies included cohort studies and case series reporting clinical outcomes, adverse events, recurrence rates, and hospitalization. Statistical analyses were performed with Comprehensive Meta-analysis Software and RevMan 5.

**Results:**

A total of 20 studies were included, comprising 142 patients treated with EUS-RFA and 249 with surgery. Clinical success was higher in the EUS-RFA group (97.5%) compared with surgery (88.9%). Patients undergoing EUS-RFA experienced fewer complications (23 vs. 59%), shorter hospital stays (mean 2.4 vs. 11 days), and zero procedure-related mortality. However, recurrence rates were greater with EUS-RFA (11%) than with surgery (4.8%). No significant differences were found in overall survival during follow-up.

**Conclusions:**

EUS-RFA is a safe, effective, and less invasive option for managing pancreatic insulinomas, ensuring rapid recovery and fewer complications. Nevertheless, its higher recurrence rate highlights the importance of patient selection and strict follow-up. Surgery remains the treatment of choice in resectable cases, while EUS-RFA represents a valuable alternative in high-risk or inoperable patients.

## ARTICLE HIGHLIGHTS

Endoscopic ultrasound-guided radiofrequency ablation (EUS-RFA) is a safe and minimally invasive treatment for pancreatic insulinomas.

EUS-RFA achieved higher clinical success (97.5%) and fewer complications compared with surgery.

Surgery remains the treatment of choice for resectable tumors due to its lower recurrence rate.

EUS-RFA represents a valuable alternative for high-risk or inoperable patients.

## Summary

**CENTRAL MESSAGE:**

Insulinoma is a rare neuroendocrine tumor of the pancreas, which originates in the beta cells of the pancreatic islets and produces excessive and unregulated insulin. The primary treatment for insulinoma is surgical, with the goal of tumor resection and preservation of pancreatic function. Precise preoperative localization is crucial for effective surgical management, especially with advanced imaging techniques that allow for precise intraoperative localization. Radiofrequency ablation (RFA) by endoscopic ultrasound (EUS) is an emerging technique for the treatment of pancreatic insulinomas, especially those that are not candidates for surgery or for whom a less invasive option is sought. This procedure combines the precision of EUS with the ability of radiofrequency to destroy tumor tissue through controlled heat.

**PERSPECTIVES:**

This study confirms that both surgery and EUS-RFA are effective treatment options for pancreatic insulinomas. EUS-RFA is a safe, minimally invasive alternative with high clinical success and lower complication rates, making it suitable for high-risk surgical candidates. However, due to its lower recurrence rate, surgery remains the preferred option for resectable cases. Careful patient selection is essential to optimize outcomes with either approach.

## VISUAL ABSTRACT

**Figure:**
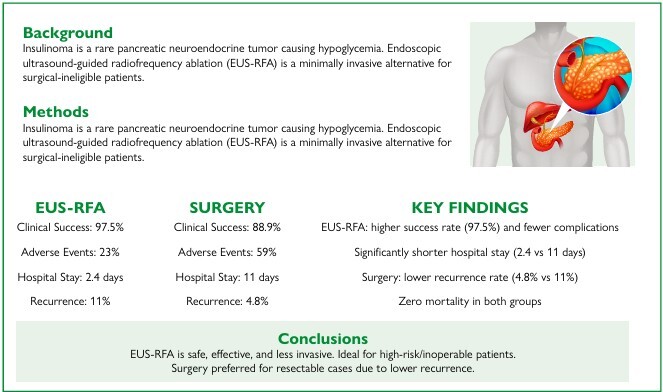

## RESUMO

**Racional:**

O insulinoma é um tumor neuroendócrino pancreático raro, originado das células beta, que leva à secreção excessiva de insulina e episódios de hipoglicemia potencialmente fatais. Embora a ressecção cirúrgica continue sendo o padrão-ouro, a ablação por radiofrequência guiada por ultrassom endoscópico (EUS-RFA) tem se destacado como uma alternativa minimamente invasiva, especialmente em pacientes não elegíveis para cirurgia.

**Objetivos:**

Metanálise conforme as diretrizes PRISMA, para comparar a eficácia e a segurança da EUS-RFA e da cirurgia no tratamento de insulinomas pancreáticos.

**Métodos:**

Foram realizadas buscas sistemáticas nas bases PubMed, Cochrane Library, Embase e Scopus, utilizando descritores MeSH relacionados a insulinoma, ablação por radiofrequência e cirurgia. Foram incluídos estudos de coorte e séries de casos com dados sobre desfechos clínicos, eventos adversos, recorrência e tempo de internação. As análises estatísticas foram feitas com os softwares Comprehensive Meta-analysis e RevMan 5.

**Resultados:**

Foram incluídos 20 estudos, envolvendo 142 pacientes tratados com EUS-RFA e 249 submetidos à cirurgia. A taxa de sucesso clínico foi maior na EUS-RFA (97,5 vs. 88,9%), com menos complicações (23 vs. 59%), menor internação (2,4 vs. 11 dias) e nenhuma mortalidade. A recorrência, porém, foi maior na EUS-RFA (11 vs. 4,8%).

**Conclusões:**

A EUS-RFA é segura, eficaz e menos invasiva, sendo alternativa valiosa para pacientes de alto risco, embora com maior taxa de recorrência. A cirurgia segue como primeira escolha em casos ressecáveis.

## INTRODUCTION

 Insulinoma is a rare neuroendocrine tumor of the pancreas, which originates in the beta cells of the pancreatic islets and produces excessive and unregulated insulin. This excess insulin production can lead to severe and potentially life-threatening hypoglycemia^36^. 

 Insulinoma is the most common functional pancreatic neuroendocrine tumor (pNET), with an incidence of 1–4 cases per million inhabitants. Metastatic insulinomas can occur in up to 10% of cases, at any stage of life, and show a slight predominance in the female sex^9,30,31^. 

 It typically occurs in adults between 30 and 60 years of age, although it can occur at any age. The most common symptoms include recurrent episodes of hypoglycemia, which may manifest as dizziness, confusion, profuse sweating, and, in severe cases, seizures or loss of consciousness. Because symptoms can be nonspecific and varied, diagnosis is often delayed^19^. 

 Diagnosis of insulinoma involves a series of biochemical and imaging tests. Fasting insulin and C-peptide levels along with the 72-h fasting test are crucial to confirm the presence of hyperinsulinism in the setting of hypoglycemia^37^. Precise tumor localization is typically achieved with imaging such as computed tomography (CT), magnetic resonance imaging (MRI), and, occasionally, glucose-labeled positron emission tomography (PET)^12^. 

 The primary treatment for insulinoma is surgical, with the goal of tumor resection and preservation of pancreatic function. Precise preoperative localization is crucial for effective surgical management, especially with advanced imaging techniques that allow for precise intraoperative localization. 

 In cases where surgery is not feasible due to tumor location or patient comorbidities, medical options such as diazoxide therapy to inhibit insulin secretion or arterial embolization as a palliative measure may be considered^31,32^. 

 Radiofrequency ablation by EUS is an emerging technique for the treatment of pancreatic insulinomas, especially those that are not candidates for surgery or for whom a less invasive option is sought. This procedure combines the precision of EUS with the ability of radiofrequency to destroy tumor tissue through controlled heat^25^. 

 Endoscopic ultrasound RFA involves insertion of an EUS probe through the gastrointestinal tract into the pancreas. This probe provides high-resolution images that allow direct visualization of the tumor. Once the insulinoma is located, a radiofrequency needle is introduced through the working channel of the endoscope and positioned in the tumor under ultrasound guidance^15^. 

 Radiofrequency is then applied to heat and destroy the tumor tissue. This process is carefully controlled to avoid damaging surrounding structures and minimize the risk of complications^8^. 

 The evidence on EUS-RFA for the treatment of insulinomas is still evolving, but preliminary studies suggest that it may be a safe and effective option for selected patients^17^. 

## METHODS

### Protocol and registration

 This study was performed according to PRISMA guidelines (Preferred Reporting Items for Systematic Reviews and Meta-Analysis) and registered in PROSPERO (International Prospective Register of Systematic Reviews) under the register CRD42024605755. 

### Search strategy

**Figure 1:**
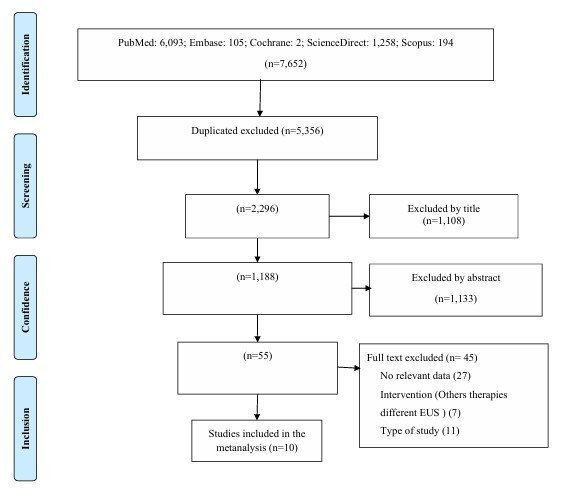
Preferred Reporting Items for Systematic Reviews and Meta-Analyses (PRISMA) flow diagram showing the study selection process, for the study group evaluating radiofrequency ablation. EUS: endoscopic ultrasound.

**Figure 2:**
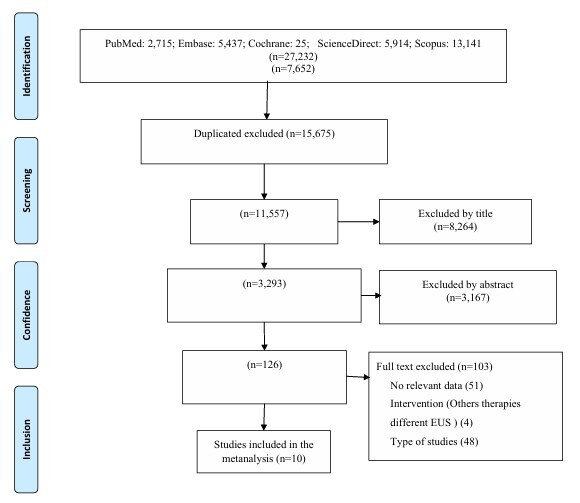
Preferred Reporting Items for Systematic Reviews and Meta-Analyses (PRISMA) flow diagram showing the study selection process, for the study group evaluating treatment with surgery. EUS: endoscopic ultrasound.

 To perform this meta-analysis, the PRISMA guidelines will be followed. Systematic searches will be carried out in the PubMed, Cochrane Library, Embase, Science Direct, and Scopus databases. Bibliography published up to June 1, 2024, will be used. The following MeSH terms will be used for the literature search: "insulinoma," "endoscopic ultrasound," "radiofrequency ablation," and "surgery." Articles in English will be searched and verified (Figure 1 and Figure 2). 

### Eligibility criteria

 The study inclusion criteria are as follows:

1. Cohort studies and case series with human patients;
2. Patients diagnosed with insulinoma treated with EUS-guided ablation and surgery; and
3. Studies in which adverse events, clinical and technical success rates, length of hospital stay, and symptom recurrence rate were reported.

The exclusion criteria are as follows:

1. Editorials, letters, reviews, meta-analyses, protocols, and case reports;
2. Detailed results were not provided or the results were unclear;
3. Insulinomas were confused with other panNETs; and
4. Duplicate studies.

Finally, a full-text check will be performed to examine whether the identified articles met the inclusion criteria. An independent researcher will perform the above processes, and the results of their search were consistent. 

### Data extraction and management

 An independent researcher extracted data from the included articles. The following data were extracted: author surname, year of publication, country of study, patient ages, number of patients with insulinoma, treatment method, adverse effects, clinical and technical success rates, length of hospital stays, and symptom recurrence rates. Clinical success is defined as the recovery of symptoms associated with insulinoma. Recurrence is defined as the reappearance of insulinoma after RFA or surgery. 

### Type of outcome measures

#### Primary outcomes

- The primary endpoints were clinical and technical success, defined by the resolution of symptoms or the resolution of the insulinoma evidenced in imaging studies during follow-up.

#### Secondary outcomes

- Assess adverse events (early or late) associated with both endoscopic and surgical procedures to analyze the safety of each approach.
- Determine intraoperative mortality related to each technique to evaluate its impact on immediate survival.
- Analyze recurrence rates of insulinomas after treatment, comparing the long-term effectiveness of both interventions.
- Compare hospital stay duration between patients undergoing EUS-guided RFA and those treated surgically, as an indicator of postoperative recovery.

### Assessment of risk of bias in included studies

 One review author (AOA) assessed the risk of bias of each included study using a tool named "non-randomized intervention studies (ROBINS-I)" for case series studies according to the recommendations in the Cochrane Handbook for Systematic Reviews of Interventions. The following definitions were used in the assessment of the risk of bias: due to confounding, arising from measurement of the exposure, in selection of participants into the study (or into the analysis), due to postexposure interventions, due to missing data, arising from measurement of the outcome, in selection of the reported result. For signaling questions within each domain for each outcome, one of the five possible answers was provided in each tool ("Yes," "Probably yes," "No," "Probably no," and "No information"), judging as "Low risk of bias," "Some concerns," or "High risk of bias." According to the algorithm result, the overall risk-of-bias judgment for each outcome was the least favorable assessment across the domains. 

### Statistical analysis

 The primary goal was to evaluate the effectiveness of the EUS-RFA technique for pancreatic insulinomas and compare it to standard surgical treatment. Secondary objectives included assessing adverse effects, procedural mortality, hospital stay duration, and recurrence rates based on imaging follow-up. Statistical analyses will be conducted using Comprehensive Meta-analysis Software (Version 4.0) and RevMan 5. A p-value of less than 0.05 will be considered statistically significant. 

### Assessment of heterogeneity

 To evaluate statistical heterogeneity, the Cochran Q test was used to assess whether observed variations in effect sizes were genuine. A p-value below 0.1 was interpreted as evidence of heterogeneity. Additionally, the I^2^ statistic was employed to quantify heterogeneity, with values interpreted as follows: less than 25% indicating no heterogeneity, 25–49% indicating low heterogeneity, 50–74% indicating moderate heterogeneity, and 75% or more indicating high heterogeneity. 

### Assessment of reporting biases

 Potential publication bias was examined by visual inspection of funnel plots. This was followed by Egger’s test to statistically evaluate asymmetry in the plots. 

## RESULTS

### Group of patients treated with radiofrequency ablation

#### Characteristics of the patients

 Ten series of studies of patients with insulinomas treated with RFA were meta-analyzed. 142 patients were included: 97 (68%) women and 45 (32%) men. The mean age was 64.2±11 years (range: 45–83 years). 142 lesions were included, with a mean size of 13.1 mm±1.8 mm (range: 1117.6 mm). The most common location of insulinomas was the head and uncinate process: 55 lesions (38.7%), neck: 5 (3.5%), body: 51 (35.9%), and tail: 31 (21.8%). Regarding the histological grade, 74% of insulinomas were Grade 1, 5.6% Grade 2, and 19.7% were not reported in the studies. The mean follow-up time was 16.87 months (ranging from 9.7 to 24 months) (Table 1). 

**Table 1:** Characteristics of patients with pancreatic insulinoma treated with radiofrequency ablation.

Author	Country	Number of patients	Middle age	Sex		Number of injuries	Tumor size	Tumor location (head and uncinate process/neck/ body/tail)	Tumor grade (G1/G2)	Follow-up	Adverse effects	Deaths	Clinical response (%)	Days of hospital stay	Recurrence
				Female	Male										
Lakhtakia et al.^23^	India	3	45	0	3	3	17.6	2/0/1/0	N/A	12	0	0	100	2	0
Oleinikov et al.^33^	Israel	7	52	4	3	7	15.04	5/0/0/2	7/0.	9.7	0	0	100	3	0
Furnica et al.^20^	Belgium	4	62	3	1	3	12.8	2/1/0/1	3/1.	22.5	2	0	100	2	0
de Nucci et al.^14^	Italy	5	80	3	2	5	12.8	0/0/3/2	5/0	12	0	0	100		0
Marx et al.^28^	France, Switzerland	7	66	6	1	7	13.3	1/3/1/2	4/1.	21	4	1	100	1.7	0
Rossi et al.^34^	Italy	3	83	1	2	3	11.6	1/0/1/1	N/A	24	0	0	100	N/A	0
Crinò et al.^11^	Italy	89	55	62	27	89	13	34/0/39/16	66/3	23	16	0	95	3	11
Borrelli de Andreis et al.^5^	Italy	10	67	7	3	10	11	3/0/3/4	9/1.	19.5	3	0	100	2	0
Debraine et al.^16^	Belgium	11	65	9	2	11	11.6	5/1/3/2	9/2.	12	0	0	80	2	2
Biermann et al.^3^	Atlanta	3	67	2	1	3	13	2/0/01	3/0.	13	1	0	100	2	0

#### Adverse events related to or treatment

 There were 25 patients with adverse effects: 11 patients with pancreatitis, nine with abdominal pain, two with bleeding, one with splenic hematoma, one with intestinal perforation, and one with retrogastric collection. 

**Figure 3:**
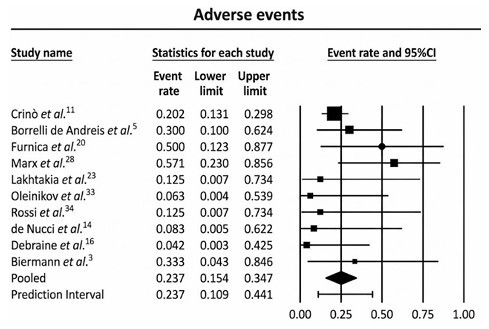
Adverse events in the pancreatic insulinoma radiofrequency ablation group. CI: confidence interval.

 The combined adverse events in patients undergoing RFA of pancreatic insulinomas were 23% (Figure 3). 

#### Mortality rate

**Figure 4:**
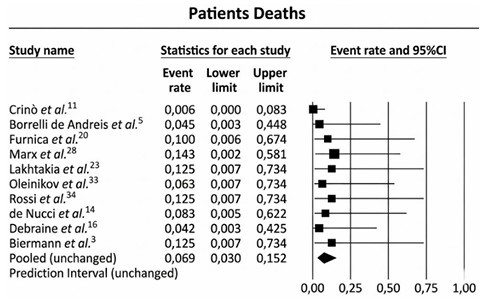
Cumulative mortality in patients treated with radiofrequency ablation. CI: confidence interval.

 The mortality rate of the procedure was zero (0%). The overall mortality rate over time was 6.9% (Figure 4). 

#### Technical and clinical success rate

 The technical success rate, defined as the absence of insulinoma recurrence on imaging examinations performed during follow-up, was 90.84%. The clinical success rate was 97.5%. 

#### Recurrence

**Figure 5:**
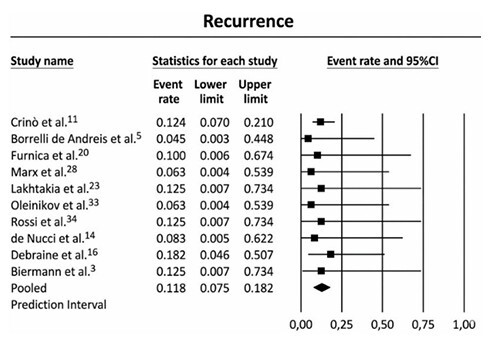
Recurrence rate in patients treated with radiofrequency ablation. CI: confidence interval.

 The recurrence rate over the 16.87-month follow-up was 11% (Figure 5). 

#### Days of hospitalization

 Regarding post-treatment hospitalization, nine of the 10 articles reported the length of stay. The median length of stay was 2.4±0.71 days (95% confidence interval [CI]: 1.7–4). 

#### Group of patients treated with surgery

 Ten series of studies of patients with insulinomas treated with surgery were meta-analyzed, with 249 patients, including 242 who underwent surgery: 164 (66%) women and 85 (34%) men. The mean age was 43.57±8.8 years (range: 36–60 years). 275 lesions were included, with a mean size of 16.69 mm±4.12 mm (between 13 and 25 mm). 

 The most common location of insulinomas was the head and uncinate process: 102 lesions (37.09%), neck: 3 (1.09%), body: 84 (30.54%), and tail: 86 (31.27%). Regarding the histological grade, 76.6% of insulinomas were Grade 1, 6.18% Grade 2, malignant in 1.81% and 12.36% were not reported in the studies. The mean follow-up time was 16.69 months (ranging from 13 to 25 months) (Table 2). 

**Table 2:** Characteristics of patients with pancreatic insulinoma treated with surgery.

Author	Country	Number of patients	Middle age	Sex		Patients undergoing surgery	Number of injuries	Tumor size	Tumor location (head and uncinate process/neck/ body/tail)	Tumor grade (G1/G2/malignant)	Follow-up	Adverse effects	Deaths	Clinical response (%)	Days of hospital stay	Recurrence	Type of surgery
				Female	Male												
Machado et al.^26^	Brazil	59	36.3	36	23	59	79	15	0/27/23/29	55/4/0	60	31	3	98.10		2	Enucleation 22 Corpocaudal pancreatectomy 25, Corpocaudal pancreatectomy associated with enucleation 7, Duodenopancreatectomy 1
Apodaca-Torres et al.^2^	Brazil	20	37.8	12	8	20	26	25	6/0/9/11	19/0/1	60	16	0	100	16		Caudal body pancreatectomy: 2, Caudal body pancreatectomy and splenectomy: 5, Caudal pancreatectomy and splenectomy: 2, Caudal pancreatectomy+splenectomy and enucleation: 1, Enucleation: 10
Valente et al.^40^	Brazil	5	37.2	3	2	5	5	15	3/0/2/0	5/0/0	6	1	0	100	6	0	Local resection (nodulectomy)
Giraldo et al.^21^	Colombia	3	53	2	1	3	3	13	1/0/1/1	3/1/0.	Does not specify	0	0	100	5	0	Local resection: 2, Distal pancreaticoduodenectomy: 1
España-Gomez et al.^18^	Mexico	34	40	20	14	34	34	22	12/2/10/10	31/0/3	44	26	1	97	10	0	Enucleation: 9, distal pancreatectomy: 5
Bonato et al.^4^	Brazil	16	39	11	5	16	16	15	7/0/6/3	15/0/1.	5	15	0	94	18	1	Enucleations: 7, Distal pancreatectomies: 5, Subtotal pancreatectomies: 2, Corpo caudal pancreatectomies + enucleation: 2
Caldas et al.^7^	Portugal	14	60.9	12	2	7	14	14.2	9/0/3/2	6/1/0.	48	4	0	85.7		1	Enucleation: 3, Corpocaudal pancreatectomy: 2, cephalic duodenopancreatectomy: 2
Chapa-Azuela et al.^10^	Mexico	7	40	5	2	7	7	20	2/1/1/3	4/1/0 (2N/A)	32	0	0	100	N/A	0	Whipple: 1, Enucleation: 2, Distal pancreatectomy: 3, Pancreatectomy plus enucleation: 1
Torres et al.^39^	Mexico	2	37.5	2	0	2	2	14	0/0/2/0	2/0/0.	11	0	0	100	N/A	0	Distal pancreatectomy
Crinò et al.^11^	Italy	89	54	61	28	89	89	13.7	35/0/27/27	79/10/0.	37	55		100	11	0	Not reported

#### Adverse events related to or treatment

 There were 148 patients with adverse effects: pancreatic fistula: 63 (42%), biliary fistula: 1 (0.7%), common bile duct injury/stenosis: 2 (1.35%), wound infection: 9 (6%), intra-abdominal collections: 9(6%), bleeding: 1(0.7%), and not specified in 63 (42.5%) patients. 

 The combined adverse events in patients undergoing RFA of pancreatic insulinomas were 59%. 

**Figure 6:**
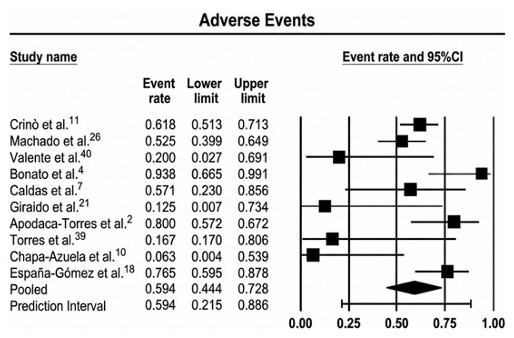
Adverse events in the pancreatic insulinoma surgical group. CI: confidence interval.

 Late complications were reported: two pancreatic pseudocysts and one incisional hernia (Figure 6). 

#### Mortality rate

**Figure 7:**
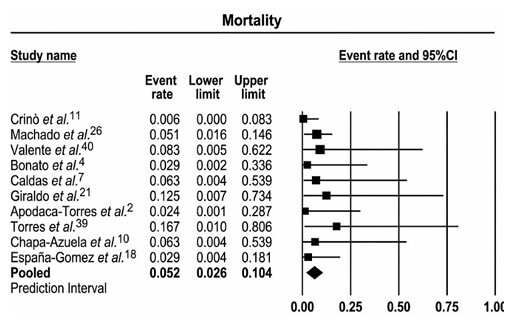
Cumulative mortality in patients treated with surgery. CI: confidence interval.

 The mortality rate of the procedure was zero (0%). The overall mortality rate over time was 5% (Figure 7). 

#### Recurrence

**Figure 8:**
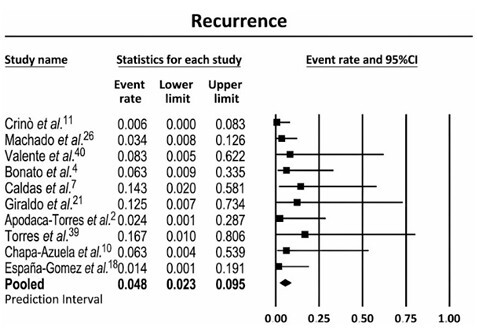
Recurrence rate in patients treated with surgery. CI: confidence interval.

 The recurrence rate over the 16.69-month follow-up was 4.8% (Figure 8). 

#### Days of hospitalization

 The median length of stay was 11 days±4.7 95%CI:5–18. 

### Comparative analysis between the group of patients treated with radiofrequency ablation and surgery

 In both groups, results related to adverse effects, clinical response, recurrence, and death during patient follow-up were analyzed. 

**Figure 9:**
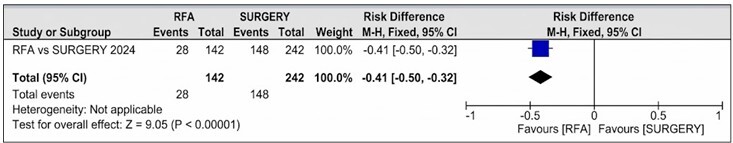
Comparison of adverse events between radiofrequency ablation and surgery. CI: confidence interval; RFA: radiofrequency ablation.

 A lower rate of adverse effects was evident in the radio-frequency group with a difference of 41% CI (0.50–0.32), p<0.05 (Figure 9). 

**Figure 10:**
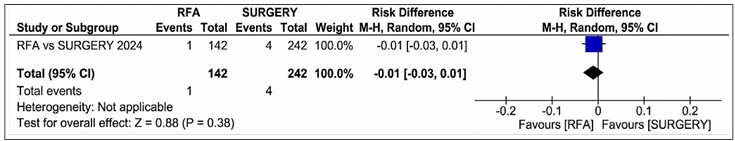
Comparison of mortality between radiofrequency ablation and surgery. CI: confidence interval; RFA: radiofrequency ablation.

 No statistically significant differences were observed between the groups in relation to overall survival during follow-up (p=0.38) (Figure 10). 

**Figure 11:**
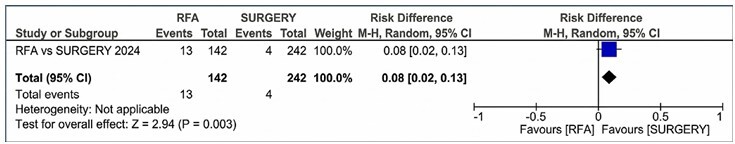
Comparison of recurrence between radiofrequency ablation and surgery. CI: confidence interval; RFA: radiofrequency ablation.

 The recurrence of lesions throughout the follow-up was statistically lower in the surgery group with a difference of 8% p<0.05 (Figure 11). 

**Figure 12:**
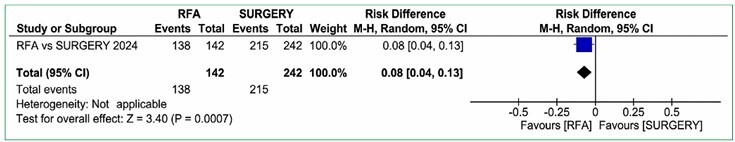
Comparison of clinical response between radiofrequency ablation and surgery. CI: confidence interval; RFA: radiofrequency ablation.

 A superior clinical response was found in the RFA group, with a difference of 8% p<0.05 (Figure 12). 

## DISCUSSION

 Pancreatic insulinoma is a rare functional neuroendocrine tumor, typically benign and solitary, arising from pancreatic beta cells and characterized by autonomous insulin secretion that causes recurrent hypoglycemia. Its incidence is low (1–4 cases per million per year), and most lesions are small (<2 cm) and manifest with neuroglycopenic and autonomic symptoms during fasting, consistent with Whipple’s triad^6,22,27,38^. Although the majority are sporadic, up to 10% are associated with multiple endocrine neoplasia type 1 (MEN1)^6,22^. 

 The primary clinical concern is severe hypoglycemia, which can be life-threatening and significantly impair quality of life, emphasizing the need for accurate diagnosis and timely treatment. Biochemical confirmation through the demonstration of hypoglycemia with endogenous hyperinsulinemia remains essential, followed by tumor localization using imaging modalities such as CT, MRI, or EUS, the latter being the most sensitive for small lesions^13,22,27,38^. 

 Surgical resection continues to be the gold standard, with cure rates exceeding 95% in most series. Enucleation is preferred for small, well-circumscribed tumors distant from the main pancreatic duct, while segmental pancreatectomy is indicated for deeper or less accessible lesions^6,13^. Despite its effectiveness, surgery carries significant morbidity, including pancreatic fistula and other postoperative complications, reported in up to 72% of cases^1,13^. 

 Over the past decade, EUS-RFA has emerged as a minimally invasive alternative for patients unfit for surgery or unwilling to undergo it. The pooled data from recent studies demonstrate high technical and clinical success rates (95100%), a lower incidence of severe adverse events (1–18%), and significantly shorter hospital stays (2–3 days compared to 7–11 days for surgery). However, EUS-RFA is associated with a higher rate of local recurrence (15–17% vs. 1–3% after surgery)^5,28,29,35,41^. These findings suggest that while EUS-RFA provides excellent short-term efficacy, long-term durability remains to be determined, underscoring the need for extended follow-up and well-designed comparative trials^5,11^. 

 Overall, the results of this meta-analysis support surgery as the standard treatment for resectable insulinomas, ensuring definitive management in most cases. Nonetheless, EUSRFA represents a promising and safe therapeutic option in selected patients, particularly those with significant comorbidities or high surgical risk. The balance between invasiveness, morbidity, and recurrence risk should guide individualized treatment decisions^1,6,11,13,27-29,35,38,41^. 

## CONCLUSIONS

 Endoscopic ultrasound-guided radiofrequency ablation (EUS-RFA) is a safe, effective, and minimally invasive therapeutic option for the management of pancreatic insulinomas, demonstrating high clinical success rates, fewer adverse events, and shorter hospital stays compared to surgical treatment. These findings support its role as an attractive alternative, particularly in patients with significant comorbidities or high surgical risk. 

 However, EUS-RFA is associated with higher recurrence rates, underscoring the importance of careful patient selection, standardized treatment protocols, and strict long-term follow-up. Surgical resection remains the gold standard for resectable insulinomas due to its lower recurrence rates and more durable outcomes. 

 Importantly, the current evidence is based mainly on retrospective studies and case series, highlighting the need for prospective, well-designed comparative trials with longer follow-up to better define the long-term efficacy and optimal indications of EUS-RFA. 

 Therefore, treatment strategies should be individualized, considering tumor characteristics, patient comorbidities, and institutional expertise, to achieve the best clinical outcomes. 

## Data Availability

The information regarding the investigation, methodology and data analysis of the article is archived under the responsibility of the authors.
